# Acute Isolated Irreducible Proximal Interphalangeal Joint Dislocation of the Fifth Toe in a Toddler: A Case Report

**DOI:** 10.7759/cureus.25630

**Published:** 2022-06-03

**Authors:** Neeraj Vij, Mohan Belthur, Ashish S Ranade

**Affiliations:** 1 Department of Orthopedic Surgery, University of Arizona College of Medicine - Phoenix, Phoenix, USA; 2 Pediatric Orthopedics, Phoenix Children's Hospital, University of Arizona College of Medicine - Phoenix, Phoenix, USA; 3 Blooming Buds Centre for Pediatric Orthopedics, Deenanath Mangeshkar Hospital and Research Centre, Pune, IND

**Keywords:** surgical stabilization, foot and ankle, pediatric trauma, open reduction and pin fixation, pipj dislocation

## Abstract

A male child aged three years and three months presented after stubbing his right fifth toe. Imaging revealed a dorsolateral dislocation of the proximal interphalangeal joint (PIPJ). After failed attempts at closed reduction, open reduction and internal fixation was pursued. At the one-year follow-up, the patient was found to be doing well clinically and radiographically. These types of injuries require a high degree of clinical suspicion to obtain the proper imaging. The interposition of adjacent soft tissues can render these injuries irreducible. When irreducible, open reduction and pin fixation may be appropriate after an adequate trial of closed reduction under anesthesia. Concomitant ligamentous injuries, avulsion injuries, and fracture-dislocations often accompany these injuries; however, they can also occur in isolation.

## Introduction

Acute toe proximal interphalangeal joint (PIPJ) dislocations are rare entities. Even though they are usually traumatic, a variety of injury mechanisms have been described [1-3]. Closed reduction is often successful in treating these injuries [4,5]. However, due to the interposition of the surrounding soft tissues, PIPJ dislocations may become irreducible. Such cases generally require a temporary pin arthrodesis. Open reduction and internal fixation [3,6] or ligament repair [1] may also be performed in cases of fracture-dislocation [3,6] or ligamentous injury [1].

Interphalangeal dislocations of the great toe are extensively studied in the literature [7-10]. However, the literature on PIPJ dislocations is sparse and is limited to case reports [1-6,11-16], with only one case series on chronic dislocations in adults [4]. Very few of these reports describe PIPJ dislocations in children [1-3]. Two of these describe dislocations of the second [1] and fourth toe [3] and one of these describes a chronic missed fifth toe fracture-dislocation [2]. Furthermore, the published reports all involve concurrent ligamentous or bony injury [1-3]. In this report, we present an unusual case of an isolated acute irreducible dorsolateral PIPJ dislocation in the fifth toe of a toddler.

The family of the patient provided their permission for submitting the data concerning the case for publication.

## Case presentation

History and physical examination

We present a case of a male child aged three years and three months with acute right-sided fifth toe pain and swelling. The patient’s family reported the child stubbing the toe against the ground while playing. Closed reduction at an outside facility had been unsuccessful.

**Figure 1:**
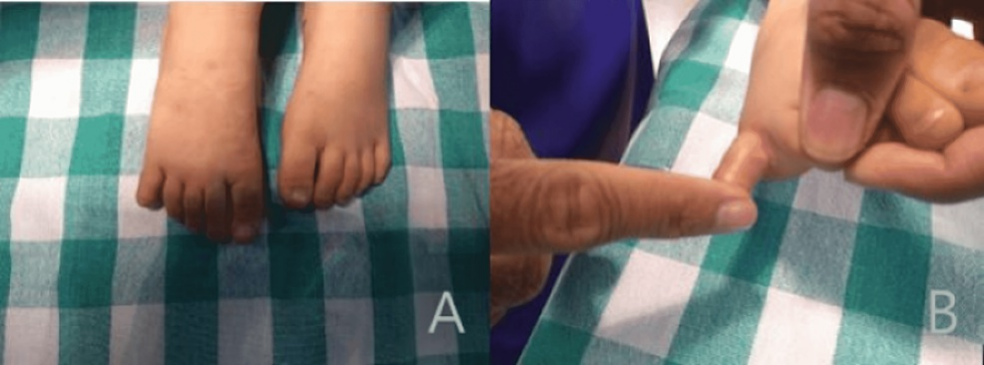
Clinical photographs on initial presentation Clinical photographs demonstrate a swollen and mildly abducted left toe (panel A) with hyperabduction of the fifth proximal interphalangeal joint after maximum metatarsophalangeal joint abduction (panel B)

Examination revealed swelling, a mild abduction deformity, and tenderness over the proximal phalanx (Figure 1).

The patient had a limited range of motion as compared to the contralateral left fifth toe. Lower extremity sensation and pulses were normal. The skin was intact.

Imaging

**Figure 2:**
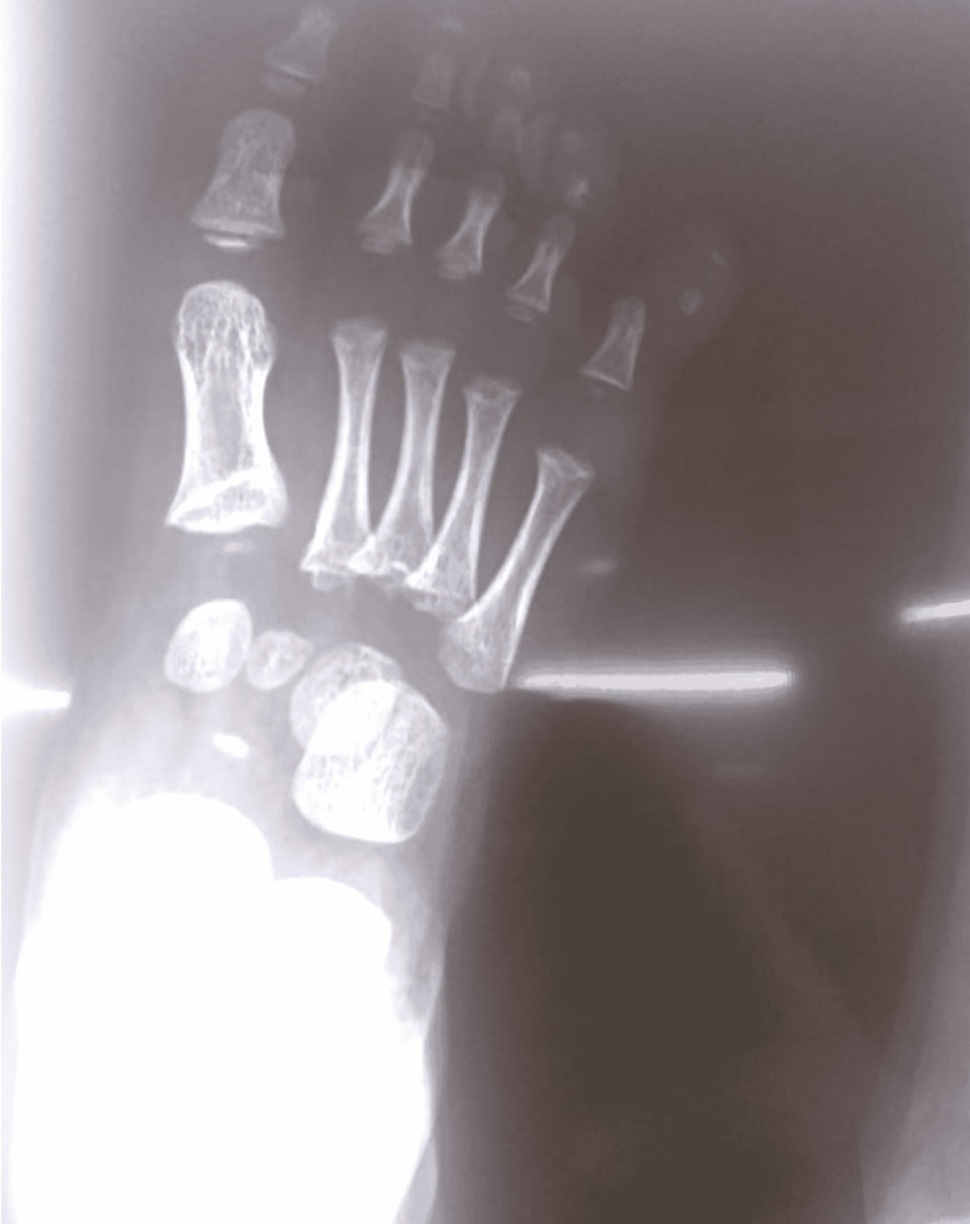
Radiograph on initial presentation Anteroposterior radiograph of the foot at initial presentation demonstrating a dorsolateral dislocation of the fifth digit proximal interphalangeal joint

Imaging from the outside facility was reviewed. Anteroposterior (AP) radiograph of the right foot demonstrated a dorsolateral dislocation of the right fifth PIPJ (Figure 2).

Treatment

The patient failed closed reduction in the emergency department. The family was counseled that an attempt at closed reduction under anesthesia was the next reasonable option. They were also informed that in the event of a failed second attempt at closed reduction under anesthesia, open reduction and internal fixation via a Kirschner wire (K-wire) would be appropriate. After understanding the risks and benefits of both these procedures, the family elected to proceed.

**Figure 3:**
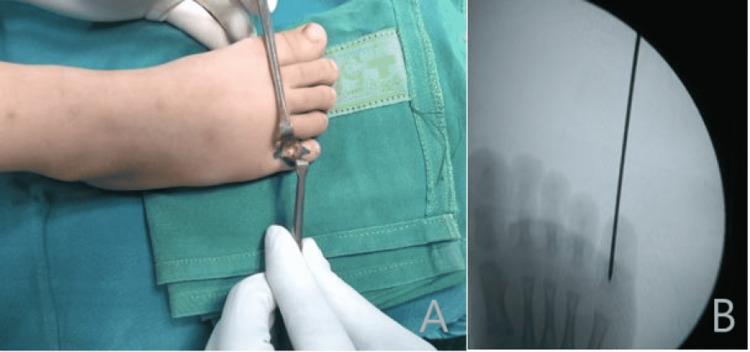
Intraoperative photograph and radiograph Exposure of the dorsal aspect of the proximal interphalangeal joint (panel A) and anatomic reduction of the fifth digit proximal interphalangeal joint with Kirschner wire stabilization (panel B)

A second attempt at closed reduction was made in the operating room. However, this was unsuccessful too. At this time, the decision was made to proceed with an open reduction. A dorsal incision over the right fifth PIPJ was made. No concurrent fracture was visualized. However, there was obvious interposition of the plantar plate and torn capsule on the plantar side of the joint, preventing reduction. The entrapped soft tissues were removed, and the joint was appropriately reduced. At this point, the range of motion and stability of the joint were reevaluated. Due to residual instability, the fifth PIPJ was stabilized with one 1.2-mm K-wire (Figure 3).

The skin was closed in the usual fashion and placed into a short leg non-weight-bearing cast with a toe plate.

Follow-up and outcome

At three weeks postoperatively, the patient reported no pain. At this point, the cast and pin were removed. There was no pin-site erythema or tenderness. The fifth toe PIPJ demonstrated good clinical alignment and the range of motion was within five degrees as compared to the contralateral side.

**Figure 4:**
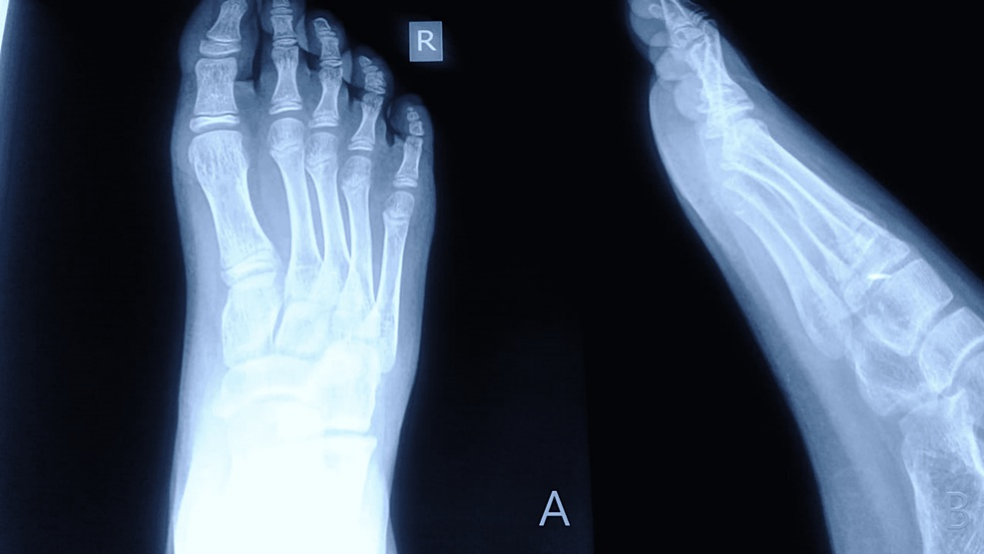
Radiographs at the one-year follow-up Anteroposterior (panel A) and lateral (panel B) radiographs of the foot one year postoperatively demonstrate maintained articular congruity of the fifth proximal interphalangeal joint

One year postoperatively, the patient was continuing to do well and had no pain. He had returned to full pre-injury weight-bearing activities. Clinically and radiographically, the correction was maintained (Figure 4).

## Discussion

The literature regarding PIPJ dislocations in children is sparse and is limited to case reports [1-3]. The published cases in the literature generally involve a concurrent bony or ligamentous injury. These include a concomitant fracture-dislocation [2], medial collateral ligament injury [1], or avulsion injury [3]. Kushare et al. [2] have described a chronic fracture-dislocation that led to a progressive deformity requiring treatment. In contrast, we presented an isolated acute irreducible dorsolateral PIPJ dislocation that responded well to surgical management.

Acute interphalangeal dislocations have been widely studied in adults [4,5]. In a case series of 18 adults, more than half of the patients were successfully treated using closed reduction with less than half of those requiring general anesthesia [5]. However, there are no large-scale studies of this injury in children in the literature [1-3]. Moreover, there are no guidelines or recommendations regarding the timing of imaging and intervention for these injuries.

The management of acute fifth toe PIPJ dislocation in children can be tricky given the lack of familiarity with this injury. Due to the interposition of the surrounding soft tissues, this injury requires a careful dissection to adequately reduce the joint. Interposed tissues may include the plantar plate [2,3] or the collateral ligaments [1,17]. In our case, the dorsal approach provided enough exposure to adequately reduce the joint.

In children, it is important to consider other concomitant injuries that may need to be addressed at the time of surgery. An avulsion fracture can occur considering the difference in strength between ligaments and the cartilaginous physis, which can be reduced with an additional pin [3]. In rare cases, the operative assessment may demonstrate the need for a ligamentous repair [3]. Suture anchors are a good option when ligamentous instability is suspected [3]; however, this was not necessary in our case.

## Conclusions

Our case demonstrates a few teaching points. Firstly, acute fifth toe dislocations can be overlooked given the swelling and lack of obvious deformity. A high index of clinical suspicion for this injury is required in order to obtain appropriate AP, lateral, and oblique radiographs of the PIPJ. Secondly, these injuries may be irreducible given the interposition of the plantar plate and/or flexor tendons. When irreducible, closed reduction under anesthesia should be trialed with the appropriate distraction and relocation maneuver. If this fails, open reduction and pin fixation is a viable treatment option, which led to good clinical and radiographic results in our patients. Finally, though often associated with concurrent bony or ligamentous injuries, these injuries can also occur in isolation as in our case.
